# Psychosocial Aspects of Caregiving That Influence Stress and Burden Among Parents of Children With Type 1 Diabetes

**DOI:** 10.1002/nop2.70452

**Published:** 2026-03-09

**Authors:** Å. Carlsund, Å. Hörnsten, U. Isaksson

**Affiliations:** ^1^ Department of Nursing Umeå University Umeå Sweden

**Keywords:** nursing, parental burden, parental stress, psychosocial aspects, type 1 diabetes

## Abstract

**Aim:**

This study aimed to examine the stress and burden experienced by parents of children with type 1 diabetes (T1D).

**Design:**

A quantitative cross‐sectional approach was used, including the results from an online questionnaire about stress and burden in parents of children (10–17 years) with T1D.

**Methods:**

The data were collected using the Swedish‐translated version of the Parental Stress Scale and the Zarit Scale of Caregiver Burden, analysed and presented through descriptive and inferential statistics.

**Results:**

Parents with a university education reported lower stress, burden, role strain, and personal strain but higher satisfaction compared to those with a high school education. Cohabiting participants reported lower stress than singles (*d* = 0.301), though these differences were not statistically significant. No significant differences were found regarding age, number of children, or place of living. Satisfaction was negatively correlated with stress, personal strain, and role strain, while stress was positively correlated with personal and role strain.

**Conclusion:**

Parents with higher education and greater caregiving satisfaction reported lower stress, while single parents and those experiencing high role strain were most vulnerable. These findings highlight the importance of family‐centered interventions and accessible psychosocial support to reduce stress and enhance outcomes for children with T1D.

**Patient or Public Contribution:**

The findings highlight the importance of identifying parents who need support in medical, educational, emotional, and psychological areas. Healthcare professionals must implement family‐centered interventions to create a supportive environment and enhance health outcomes for the entire family.

## 1 Introduction

Parents play a crucial role in their children's lives and daily experiences, influencing their cognitive, educational, psychosocial, and emotional development (Vahedi et al. 2019). Despite its significance, parenting is a challenging and demanding responsibility, with the evolution of family life introducing new challenges for parents (Nomaguchi and Milkie 2020). Parents of healthy children experience typical yet sometimes stressful and demanding developmental phases in their child, marked by hormonal fluctuations and a growing sense of autonomy. These phases may become even more complex when children seek independence while managing a chronic illness, such as Type 1 Diabetes (T1D) (Hamilton et al. 2017, 2021; Rybak et al. 2017; Stefanowicz et al. 2018). Type 1 Diabetes is a lifelong autoimmune disease managed with insulin injections via pen or pump. The cause of T1D is not fully understood, but genetic factors, environmental influences, and certain viruses seem to contribute (Cerolsaletti et al. 2019; Craig et al. 2019).

Transitioning from childhood to adolescence can be particularly challenging in families where a child has T1D, necessitating a supportive family environment to reduce parental stress and promote overall well‐being (Rybak et al. 2017). A lack of time and energy can result in stress and burden for parents (Azimi et al. 2024; Khemakhem et al. 2020; Patton et al. 2016). Previous research indicates that parents of children with T1D experience increased stress compared to parents of healthy children (de Maat et al. 2021; Smith et al. 2015). Research shows that parents sometimes prioritise the well‐being of their sick child over their own health (Rifshana et al. 2017). For example, caring for a child with T1D can affect parents' ability to maintain relationships with family and friends and negatively impact their work, home responsibilities, self‐care, and finances (Khemakhem et al. 2020; Shulhan‐Kilroy et al. 2022). In the context of T1D caregiving, stress and burden are related but distinct concepts. Stress includes emotional and psychological challenges, such as fears related to hypo‐ and hyperglycemia, carbohydrate counting, technical aids, and long‐term complications (Liu et al. 2022; Yi‐Frazier et al. 2024). Burden, on the other hand, refers to the overall impact on various aspects of parents' lives, including time, energy, lack of support, and social isolation (Toledano‐Toledano and Domínguez‐Guedea 2019; Toledano‐Toledano and Luna 2020). In summary, while stress describes the immediate emotional responses to specific challenges, burden reflects the cumulative and ongoing effect of caregiving on parents' overall well‐being and daily life. Both concepts are essential to understanding the experiences of parents caring for a child with T1D, but they capture different aspects of the caregiving experience.

Stress and burden can significantly impact parents' well‐being and their ability to care for their children effectively (Azimi et al. 2024; Shattnawi et al. 2023). Emotional and psychological challenges such as fears about hypo‐ or hyperglycemia, carbohydrate counting, dependence on technical aids, and anxiety about long‐term complications are common sources of stress. The ongoing stress of living with a chronic illness can also affect the family environment and long‐term health (Li and Liu 2020; Wang et al. 2024). Furthermore, how families handle conflicts is vital for children's diabetes self‐management, with increased family conflict associated with poorer glycemic control. More frequent and less effectively managed conflicts are connected to poor treatment adherence and children's well‐being in T1D (DeCosta et al. 2020; Farthing, Bally, Rennie, et al. 2022; Farthing, Bally, Leurer, et al. 2022), which can negatively influence parental stress and burden (Azimi et al. 2024; Shattnawi et al. 2023; Theofilou and Vlastos 2023).

Understanding whether and how parents are affected by their child's T1D is essential for the family's short‐term and long‐term well‐being (Kimbell et al. 2021; Liu et al. 2022; Patton et al. 2023). However, few studies have explored the connection between family demographics and parental stress in Swedish families with T1D or validated stress and burden scales specifically for this group. Recent Swedish research has validated the Parental Stress Scale (PSS) for use among parents of children with disabilities, showing good reliability and validity in Swedish settings (Lindström et al. 2024), but studies focusing solely on parents of children with T1D are still limited. Additionally, qualitative research from Sweden and Finland emphasises the need for a deeper understanding of how family transitions, daily routines, and support systems influence parental well‐being (Carlsund et al. 2025; Pironetti et al. 2025). There is also a call for studies that investigate how psychosocial interventions, and social support can reduce stress and burden in these parents (Marker et al. 2021). This study aims to fill these gaps by examining stress and burden among parents of children with T1D in Sweden, with particular attention to demographic factors such as cohabitation status, and by using Swedish‐validated tools to measure parental stress. By focusing on a Swedish sample and including both mothers and fathers, this research provides new insights into the factors that influence parental well‐being in families affected by T1D. Therefore, this study aims to assess the stress and burden experienced by parents of children with T1D.

## 2 Theoretical Framework

Bronfenbrenner's ecological systems theory offers an analytical framework for understanding how an individual's development occurs within a context of multiple interacting system levels. The theory is particularly relevant for analysing parents' experiences of caring for a child with type 1 diabetes (T1D), as this situation involves complex interactions between the different system levels (Bronfenbrenner 1979). The *microsystem* encompasses the child's closest relationships, where parents play a central role in managing illness. Parents are responsible for daily care, blood sugar monitoring, and insulin administration, which requires a high degree of commitment and competence. This responsibility can increase psychological stress and affect parents' well‐being. The *mesosystem* consists of the relationships between the parts of the microsystem, such as the connection between family and work. When the caregiving responsibility increases, parents may find it difficult to maintain social relationships or work engagement, which increases stress. This demonstrates how changes in the microsystem (the child's care needs) can create chain reactions in the mesosystem (parents' social networks and work). The *exosystem* includes structures that indirectly affect the family, such as working conditions and access to support resources. Parents with higher education can more easily navigate the healthcare system and absorb information, which reduces stress. Conversely, lower education and socioeconomic status can limit resources and create communication barriers with healthcare professionals (Neyra Marklund et al. 2022). The *macrosystem* encompasses cultural and societal norms, including how care is organised and the expectations placed on the parental role. In Sweden, there is structured support within diabetes care, but despite this, differences in education and socioeconomic status can lead to inequalities in how the support is utilised (Bronfenbrenner 1979; Khemakhem et al. 2020).

## 3 Methods

### 3.1 Aim

This study aimed to examine the stress and burden experienced by parents of children with T1D.

### 3.2 Design

A quantitative cross‐sectional approach was used, including the results from an online questionnaire about stress and burden in parents of children (10–17 years) living with T1D. A quantitative approach is appropriate for measuring numerical data from structured questionnaires, which can be used in future studies to compare results over time or between populations. Furthermore, online data collection is fast and cost‐effective (Cathala and Moorley 2018). The study is part of a broader research project exploring parental experiences in caring for a child with T1D. In the initial phase, we conducted qualitative interviews with parents to gain a detailed understanding of their lived experiences. These interviews helped select the questionnaires used in the current study, ensuring that the items addressed relevant aspects of stress, caregiving burden, and satisfaction identified from the qualitative data.

### 3.3 Participants and Procedure

Determined on ease of access and to gain in depth insights from parents with specific experience of T1D, participants were recruited through purposive sampling (Shorten and Moorley 2014). The presumptive participants were systematically sought via four closed Swedish Facebook groups for parents of children with T1D, where membership required answering diabetes‐related questions and approval from group administrators (Garner et al. 2023). Although independent verification of parental status or the child's diagnosis was not possible, the group's admission process and focus provided reasonable assurance that the inclusion criteria were met. Parents provided informed consent before completing the questionnaire. Descriptions of the participants are presented in Table 1. As mentioned above, potential participants were invited via posts on closed Facebook groups. The post included informed consent, the purpose of the study, the Swedish translation of the ‘Parental Stress Scale’ (PSS), the Swedish translation of the ‘Zarit Scale of Caregiver Burden,’ and contact information for the research group. If the presumptive participants provided informed consent, they were asked to complete the questionnaires. Eligibility was operationalised in the recruitment posts by clearly stating that only parents or caregivers of children aged 10–17 years with T1D could participate. This was reinforced by posting in closed Facebook groups where membership required confirmation of T1D experience. The data were processed in accordance with the General Data Protection Regulation (GDPR 2016/679), and each answer was labelled with a number. The participants' informed consent and questionnaire responses were recorded in Research Electronic Data Capture (RedCap), a secure data collection tool at Umeå University. The demographic data collected in the online questionnaire included age, sex, education, marital status, number of children, and place of living.

**TABLE 1:** Descriptions of the participants (*n* = 90).

Age (*m*, SD)	45.17 (5.97)
Sex (*n*, %)	
Women	84 (93.3)
Men	6 (6.7)
Education (*n*, %)	
High School	18 (20.0)
University	72 (80.0)
Marital status (*n*, %)	
Cohabiting	71 (78.9)
Single	19 (21.1)
No. of children (*n*, %)	
One	31 (34.4)
Two	36 (40.0)
Three or more	23 (25.6)
Place of living (*n*, %)	
Urban	52 (57.8)
Rural	31 (34.4)
Remote area	6 (6.7)
Other	1 (1.1)

### 3.4 Questionnaires

The data were collected using the Swedish‐translated version of the Parental Stress Scale and the Zarit Scale of Caregiver Burden. The Parental Stress Scale (PSS), developed by Berry and Jones in 1995, is an 18‐item instrument designed to assess parental stress and satisfaction (Berry and Jones 1995). The PSS is valid and reliable for parents of children with chronic health conditions (Zelman and Ferro 2018). Previous factor analysis revealed a four‐factor structure comprising two negative factors (Parental Stressors and Lack of Control) and two positive factors (Parental Reward and Parental Satisfaction) (Berry and Jones 1995). However, Harding et al. (2020) identified a two‐factor solution that more effectively captured stress and satisfaction related to the parenting role. The PSS employs a 5‐point Likert scale, ranging from 1 (strongly disagree) to 5 (strongly agree), to measure parental stress and satisfaction. Scores are computed by adding all items, resulting in a satisfaction subscale ranging from 7 to 35 and a stress subscale ranging from 9 to 45. Higher scores reflect increased parental satisfaction or stress (Berry and Jones 1995; Harding et al. 2020). In the PSS, s*atisfaction* includes social support, marital, and job satisfaction, while *stress* includes demands, worries, and anxiety. Two authors (UI & ÅH) translated the instrument from English to Swedish, which was then translated back to English by an authorised translator, compared, slightly adjusted, and subsequently accepted by the instrument developer.

The Zarit Scale of Caregiver Burden, also known as the Zarit Burden Interview, is a widely used questionnaire developed by Zarit et al. (1980). Initially designed with 29 items, it was later reduced to 22 questions. The scale was initially developed to assess the subjective feelings of burden experienced by caregivers of older individuals with dementia and other disabilities. It has later been used and viewed as valid among other caregivers, such as parents of children with T1D (Bilgehan et al. 2024). The questionnaire employs a 5‐point Likert Scale, ranging from 0 (never) to 4 (nearly always). The total score ranges from 0 to 88, where scores of 0–21 indicate no to mild burden, 21–40 indicate mild to moderate *burden*, 41–60 indicate moderate to severe burden, and scores of 61 or higher indicate severe burden (Zarit et al. 1980). The total score has later been separated into two subscales: *personal strain* (score 0–48), that is, how personally stressful the experience of caregiving is, and *role strain* (score 0–24), that is, the stress due to role conflict or overload (Whitlatch et al. 1991). The questionnaire was previously translated into Swedish, and a user agreement was established between Mapi Research Trust (MR) and the first author (ÅC) (No. 105121). The original developer was contacted via email and suggested replacing the term ‘relative’ with ‘child’ to better fit the context for parents and their caregivers' children. This linguistic adaptation was used in research with the Zarit Scale of Caregiver Burden in healthcare settings like caring for children with chronic illnesses or disabilities (Bhusal et al. 2025; Domínguez‐Vergara et al. 2023).

### 3.5 Data Analysis

#### 3.5.1 Statistics

The quantitative data were analysed and presented using descriptive and inferential statistics. Independent *t*‐tests and ANOVA were used to compare differences between different demographic variables. Shapiro‐Wilks test was used to test for normality. The effect size was calculated using Cohen's *d* for *t*‐test or partial eta‐square (*ɳ* ^2^ *p*) for ANOVA. According to Cohen (2013), benchmarks for the effect size for *d* are 0.2, 0.5, and 0.8, respectively, for small, medium, and large effect sizes, and *ɳ* ^2^ *p* are 0.01, 0.06 and 0.14, respectively (Cohen 2013). Persons' correlation analysis tested correlations between stress, satisfaction, role strain and personal strain. A backwards stepwise linear regression analysis with stress as the dependent variable and education, number of children, place of living, age, satisfaction, personal strain, and role strain as independent variables were conducted to analyse variables independently associated with stress. Multicollinearity was analysed using the variance inflation factor (VIF), showing values between 1.03 and 1.61 for the initial model and 1.09 to 1.69 for the final model, indicating that there was no multicollinearity present.

#### 3.5.2 Rigour

The credibility of the study is assessed based on validity, reliability, and generalisability (Polit and Beck 2022). Validity was strengthened through the use of established and previously validated instruments, the Parental Stress Scale (PSS) and the Zarit Scale of Caregiver Burden (Berry and Jones 1995; Harding et al. 2020; Zarit et al. 1980), which have documented usefulness for parents of children with chronic illnesses (Domínguez‐Vergara et al. 2023). The translation process for the PSS used a back‐translation method and was approved by the instrument's developer, which helped ensure conceptual validity. The inclusion criteria were outlined in the recruitment materials, which enhanced internal validity. Reliability was maintained by using standardised instruments with established internal consistency measures. Data collection was carried out using a secure electronic system (RedCap), which reduced registration errors and improved measurement consistency. However, generalisability is limited because the sample was recruited through closed Facebook groups using purposive sampling; participants were not randomly selected. This may impact external validity and transferability to other contexts. To enhance transparency, demographic data were reported in detail, enabling an evaluation of how well the results might be applicable to similar settings (Clark et al. 2021; Polit and Beck 2022).

### 3.6 Ethics

The Ethics Review Authority granted ethical approval (2024‐04062‐01). The research for this project complies with the Declaration of Helsinki (2013) concerning the four main requirements: information, consent, confidentiality, and utilisation of research data (World Medical Association 2013). Parents of children with T1D were asked if they agreed to participate in the project and received written information about the study's purpose and that participation was voluntary.

## 4 Results

The results regarding socio‐demographic data showed that parents with a university education rated themselves as having lower stress, burden, role strain, and personal strain, but higher satisfaction, compared to those with a high school education. There was a tendency towards a difference in stress between men and women, where men rated themselves as having lower stress (*d* = 0.214) and lower satisfaction (*d* = 0.267) compared to women. Regarding civil status, participants who cohabited reported lower stress levels than those who were single (*d* = 0.301); however, these differences were not statistically significant. No other statistically significant differences concerning age, number of children, or place of living were found (Table 2).

**TABLE 2:** Univariate associations between stress, satisfaction, and burden in parents with a child living with T1D (*n* = 124).

	Stress (range 9–45)			Satisfaction (range 7–35)			Burden (range 0–88)			Role strain (range 0–24)			Personal strain (range 0–48)		
		*p*	*d*/*ɳ* ^2^ *p*		*p*	*d*/*ɳ* ^2^ *p*		*p*	*d*/*ɳ* ^2^ *p*		*p*	*d*/*ɳ* ^2^ *p*		*p*	*d*/*ɳ* ^2^ *p*
Age (*r*) (*n* = 124)	0.026	0.821		−0.081	0.477		0.144	0.202		0.161	0.157		0.123	0.278	
Sex (*n* = 124)		0.614	0.214		0.545	0.267		0.883	0.062		0.656	0.189	0.839	0.052	
Female	27.64 (5.98)			31.74 (2.75)			37.56 (12.13)			9.64 (4.39)			18.92 (6.56)		
Male	26.33 (8.12)			31.00 (3.03)			38.33 (17.07)			10.50 (6.60)			19.67 (8.07)		
Education (*n* = 124)		< 0.001	0.905		0.085	0.339		0.006	0.739		0.005	0.757		0.021	0.353
High school	31.72 (4.98)			30.94 (2.62)			44.67 (12.51)			12.33 (4.38)			22.50 (6.87)		
University	26.51 (5.92)			31.88 (2.78)			35.85 (11.80)			9.04 (4.34)			18.18 (6.36)		
Civil state (*n* = 123)		0.247	0.301		0.635	0.142		0.582	0.143		0.941	0.019		0.617	0.076
Cohabiting	27.17 (6.19)			31.61 (2.82)			37.99 (12.41)			9.72 (4.59)			19.20 (6.54)		
Single	29.00 (5.63)			32.00 (2.58)			36.21 (12.55)			9.63 (4.36)			18.11 (7.02)		
No. of children (*n* = 124)		0.206	0.036		0.321	0.026		0.192	0.037		0.408	0.020		0.187	0.038
One	29.13 (5.83)			31.16 (3.49)			40.68 (13.98)			10.58 (4.51)			20.55 (4.51)		
Two	26.64 (5.89)			31.75 (2.36)			36.83 (12.15)			9.31 (4.99)			18.69 (4.99)		
Three or more	26.87 (6.59)			32.30 (2.12)			34.70 (9.82)			9.13 (3.70)			17.26 (3.70)		
Place of living (*n* = 124)					0.930	0.002		0.741	0.007		0.522	0.015		0.980	< 0.001
Urban	27.02 (6.27)	0.632	0.011	31.71 (3.04)			37.04 (12.84)			9.31 (4.53)			19.08 (4.53)		
Rural	28.36 (6.25)			31.81 (2.46)			39.10 (12.72)			10.48 (4.84)			19.07 (4.84)		
Remote area	27.33 (3.72)			31.33 (1.86)			36.33 (6.71)			9.50 (2.67)			18.50 (2.67)		

*Note:* *r*, correlation coefficient; *p*, *p*‐value; *d*, Cohen's *d*; *ɳ* ^2^ *p*, partial eta square.

The result also showed that satisfaction was negatively correlated with stress, personal strain, and role strain. There was furthermore a positive correlation between stress and personal strain and role strain (Table 3).

**TABLE 3:** Correlations between sociodemographic data and stress and satisfaction, burden, role strain and personal strain.

	Satisfaction	Stress	Personal strain	Role strain
Satisfaction	—			
Stress	−0.61	—		
Personal strain	−0.64	0.67	—	
Role strain	−0.59	0.72	0.73	—

A backward stepwise linear regression model was performed, where variables that showed a low to medium difference in effect size and variables that previous studies had shown to influence stress and burden were included in the initial model. The final model revealed that university education and satisfaction were independently associated with lower stress levels. In contrast, role strain was independently associated with higher stress. The final model accounted for 58% of the variation (Table 4).

**TABLE 4:** Linear regression of associations between stress, satisfaction and burden in parents with a child living with T1D.

	Initial model^a^				Final model^b^			
	*B*	*β*	CI	*p*	*B*	*β*	CI	*p*
Education								
University—High school	−2.03	−0.33	−4.31 to 0.25	0.081	−2.41	−0.40	−4.58 to 0.25	0.029
No. of children								
Two—One	−1.44	−0.24	−3.71 to 0.83	0.209				
Three or more—One	−1.16	−0.19	−3.96 to 1.64	0.410				
Place of living								
Rural—Urban	0.46	0.08	−1.50 to 2.42	0.641				
Remote area—Urban	−0.31	−0.05	−4.17 to 3.56	0.875				
Age	−0.12	−0.12	−0.29 to 0.06	0.177				
Satisfaction	−0.41	−0.19	−0.86 to 0.04	0.074	−0.59	−0.27	−0.97 to 0.21	0.003
Personal strain	0.21	0.23	−0.01 to 0.43	0.057				
Role strain	0.51	0.39	0.39 to 0.81	0.001	0.70	0.52	0.47 to 0.94	< 0.001

^a^ Initial model: adjusted *R* ^2^ = 0.57, *p* < 0.001.

^b^ Final model: adjusted *R* ^2^ = 0.58, *p* < 0.001.

## 5 Discussion

The study examined stress and burden in parents with a child living with T1D. Our results showed that parents with higher education experienced less stress than those with lower education. This may be due to the increased access to information and resources that higher education provides. However, it is important to note that while education level is often correlated with socioeconomic status (SES), they represent different demographic factors. Education refers to formal academic attainment, and SES to a broader range of indicators (Tsiouli et al. 2013) reported that families of low SES having an adolescent with diabetes were more prone to diabetes‐related stress. The children were more susceptible to poorer glycemic control, and Baharvand and Hormozi (2019) reported that parents with higher education can enhance metabolic control and provide better meal planning for adolescents with T1D (Baharvand and Hormozi 2019). Studies show children with T1D from lower‐educated families often have poorer glycemic control and well‐being than those from higher‐educated families. It has been shown that parents with lower educational attainment also have reduced health literacy, making it more challenging for them to manage diabetes tasks such as insulin adjustments and blood glucose interpretation (Kimbell et al. 2021; Neyra Marklund et al. 2022). Despite the structured support available in Swedish healthcare, less‐educated families may not fully utilise available resources (Neyra Marklund et al. 2022). Psychosocial stress is more common in these families, affecting management and routines (Patton et al. 2023). Lower education has also been found to be associated with lower socioeconomic status, limiting access to technology, healthy food, and time for care (Woodward et al. 2024). Communication barriers arise when parents feel less confident talking to healthcare professionals, leading to misunderstandings and unmet needs, despite efforts to personalise education in Sweden (Neyra Marklund et al. 2022).

A majority of participants in our study were women and university‐educated, which may have influenced the results. These demographics may limit the generalisability of the findings to the broader Swedish and international parent population of children with T1D. Although our results showed that mothers reported slightly higher stress levels than fathers, these differences were not statistically significant, and gender was not included in the regression models. Nevertheless, (Nieuwesteeg et al. 2017) found that as children age, fathers begin to take on more responsibilities for disease management, which may influence stress over time. This suggests that gender‐related differences in stress may be dynamic and context dependent, rather than consistently significant. Furthermore, (Bilgehan et al. 2024) and others report that shared parent involvement in managing a child's T1D significantly can impact disease management outcomes (Limbers and Teasdale 2018; Teasdale and Limbers 2018; Young et al. 2014), highlighting the importance of collaborative caregiving rather than focusing on gender differences alone. Our results also indicated that single parents experienced higher stress than cohabiting parents, which is in line with (Tsiouli et al. 2013), who reported that single parents experienced higher diabetes‐related stress, implying poorer glycemic balance in their children.

Our results show that higher satisfaction in the caregiving role was linked to lower experienced stress. However, Nomaguchi and Milkie (2020) highlight that, besides access to resources and support networks, parental satisfaction may stem from other aspects, such as effective parental communication and a sense of competence in managing the child's condition. A recent dissertation emphasises the crucial roles that social support networks and access to resources play in alleviating stress and boosting parenting self‐efficacy for parents of children with T1D (Carroll 2021). Carosi Arcangeli et al. (2024) have also reported that parents of children with T1D exhibited higher levels of mental health issues compared to parents of children without chronic diseases (Carosi Arcangeli et al. 2024). Therefore, it is essential for healthcare professionals working with this group of families to recognise the importance of providing supportive resources. Providing support and encouragement to parents and caregivers is crucial for mitigating potential adverse mental health effects on children with T1D and their parents.

Our results showed that the greater the burden experienced by parents of children with T1D, the higher the levels of stress they tended to face. However, it is positive that as children grow older, parents experience decreased daily stress (Nieuwesteeg et al. 2017). This is important since parental diabetes‐specific distress predicts an increase in children's depressive symptoms (Bassi et al. 2020; Patton et al. 2023). Parental stress also predicts deterioration in HbA1c levels (Tsiouli et al. 2013). On the other hand, high parental self‐efficacy is associated with better monitoring, which leads to improved adherence and more balanced HbA1c levels in their children (Bassi et al. 2020). Nevertheless, the emotional burden persists, and Saßmann et al. (2022) recommend providing ongoing support for parents through, for instance, easily accessible support for mental health concerns and practical skills (Saßmann et al. 2022).

Our findings underscore the necessity of offering comprehensive support to parents of children with T1D. This support encompasses not only medical and educational resources but also emotional and psychological assistance to enhance parental satisfaction and alleviate stress. Healthcare providers should consider implementing family‐centred interventions that cater to the needs of both the parents and their children (Ispriantari et al. 2023; Nieuwesteeg et al. 2017), cultivating a supportive environment that encourages better health outcomes for the entire family (Rostaminasab et al. 2023).

Viewed through Bronfenbrenner's ecological systems theory, these findings show that interactions across multiple system levels influence parental stress. At the microsystem level, greater satisfaction with the caregiving role appears to act as a protective factor, reducing stress within the immediate parent–child relationship. The higher stress levels among single parents highlight vulnerabilities in the mesosystem, where limited social and relational support increase caregiving difficulties. Education‐related differences reflect the impact of the exosystem: parents with higher education can better navigate healthcare systems and access resources. In comparison, lower education and socioeconomic status limit these opportunities and create communication barriers (Neyra Marklund et al. 2022). Despite Sweden's well‐organised healthcare support, ongoing disparities indicate limitations within the macrosystem, suggesting that universal services alone cannot eliminate inequalities. Overall, these results emphasise that parental stress is not an isolated issue but a result of complex interactions across ecological systems. Protective factors, such as access to educational resources and positive caregiving experiences, can help reduce stress. At the same time, vulnerabilities associated with single parenthood and lower educational levels suggest areas for targeted support. This view highlights the importance of family‐centered approaches that go beyond medical care to include psychosocial support and fair resource distribution, ultimately aiming to improve health outcomes for both children and their families.

The choice of a quantitative design was justified by the study's aim to measure the prevalence and variation of stress, satisfaction, and burden among parents and to examine associations with demographic and psychosocial factors. Quantitative methods allow for systematic measurement and statistical analysis of these relationships, which is essential for identifying patterns that can inform targeted interventions. Furthermore, the use of structured questionnaires enables comparability across populations and over time, supporting future longitudinal or cross‐cultural research. Online data collection was selected for its efficiency and cost‐effectiveness, as well as its ability to reach a geographically dispersed population (Cathala and Moorley 2018).

This study has some limitations. When we started our research, we were unaware of another Swedish translation of the Parental Stress Scale (PSS). This became clear in early 2025 after we had already translated the scale, been in contact with the main developer, and used the scale in a project. We contacted the authors of the other translation (Lindström et al. 2024) and reviewed their version. Upon comparison, we found that the translations were quite similar. Although our Swedish version of the Parental Stress Scale (PSS) is similar to Lindström et al.'s (2024) version, we have not conducted a detailed comparison of individual questions. The similarity suggests that the content is essentially the same, but since we have not directly compared them, there may be minor differences in wording or question structure. These differences could influence how parents interpret and respond to specific questions, especially if some questions are sensitive to cultural or linguistic nuances. This could impact the interpretation of the results if such differences exist. To ensure that results are comparable and valid across different Swedish versions of the PSS, future research should analyse individual questions and factor structures. This will strengthen evidence that the PSS is effective for Swedish parents and that findings are consistent across studies.

Secondly, we have not used any cut‐off point for high and low caregiver burden using the Zarit Scale. According to the user manual, identifying a single universal cut‐off point for the risks associated with high caregiver burden is not practical. This is because the risks can vary significantly among caregivers. Being ‘at risk’ can encompass different meanings, such as experiencing increasing depression, declining health, suicidal or homicidal thoughts, or engaging in physical or abusive behavior. Furthermore, the type of risk may differ from one caregiver to another. Additionally, the translated version of the Zarit Scale was not pilot tested. This decision was based on previous research showing that similar linguistic adaptations of the scale have been successfully used in other healthcare settings, such as in studies with children who have chronic illnesses or disabilities. These studies suggest that the scale can withstand translation and contextual changes, and that the instrument remains valid even without separate pilot testing (Bhusal et al. 2025; Domínguez‐Vergara et al. 2023). Furthermore, we were in contact with Zarit, who proposed and approved that we replace the word ‘relative’ with ‘child’.

The variables selected for the regression model were based on previous research that identified factors such as education, number of children, place of residence, age, satisfaction, and caregiver strain as relevant to parental stress in families with T1D (Bassi et al. 2020, 2023; Wang et al. 2021). Marital status was included in univariate analyses but was excluded from the multivariate model because of its inconsistent association with parental stress after adjusting for other variables or to avoid multicollinearity. Studies suggest that its influence may decrease or become nonsignificant in multivariate models when other psychosocial and demographic factors are considered (Fang et al. 2024).

## 6 Conclusion

Our findings highlight the need for comprehensive support for parents of children with T1D, covering medical, educational, emotional, and psychological aspects. Healthcare providers should consider implementing family‐centered interventions to create a supportive environment that promotes better health outcomes for the entire family. Our findings showed that parents with higher education levels and satisfaction in the caregiving role experienced lower stress. Identifying parents in need of support is crucial since high caregiver burden can vary significantly among individuals. Ongoing support for parents, including easily accessible mental health interventions, is essential. Community‐based support networks, including peer support groups and counselling services, could offer parents emotional and practical assistance. Since role strain was strongly associated with parental stress in our study, targeted interventions aimed at reducing role strain are particularly important. For example, programs for sharing caregiving responsibilities could be created to support single parents, while workplace flexibility initiatives could help cohabiting parents better manage caregiving and work duties. Structured respite care and practical skills training could also ease role strain by offering parents clear and actionable strategies. More research is needed to explore the long‐term effects of family support interventions on parental stress and child health outcomes in T1D.

## Funding

This work was supported by the Swedish Diabetesfoundation.

## Ethics Statement

The Ethics Review Authority granted ethical approval (2024‐04062‐01).

## Conflicts of Interest

The authors declare no conflicts of interest.

## Data Availability

The data that support the findings of this study are available on request from the corresponding author. The data are not publicly available due to privacy or ethical restrictions.
